# Superluminal and stopped light due to mode coupling in confined hyperbolic metamaterial waveguides

**DOI:** 10.1038/srep17678

**Published:** 2015-12-08

**Authors:** Andres D. Neira, Gregory A. Wurtz, Anatoly V. Zayats

**Affiliations:** 1 Department of Physics, King’s College London, Strand, London WC2R 2LS, United Kingdom

## Abstract

Anisotropic metamaterials with hyperbolic dispersion can be used to design waveguides with unusual properties. We show that, in contrast to planar waveguides, geometric confinement leads to coupling of ordinary (forward) and extraordinary (backward) modes and formation of hybrid waveguided modes, which near the crossing point may exhibit slow, stopped or superluminal behavior accompanied by very strong group velocity dispersion. These modes can be used for designing stopped-light nanolasers for nanophotonic applications and dispersion-facilitated signal reshaping in telecom applications.

Metamaterials are media with optical properties dependent on the architecture of their subwavelength-structure. Their geometry and, thus, optical properties can be tuned to enable unusual optical phenomena such as negative refraction, cloaking, and many others^1^. Among various metamaterial designs, uniaxial anisotropic metamaterials with an elliptic or hyperbolic dispersion of the electromagnetic waves attract especial interest due to their simple realization and advantageous optical properties for sensing, nonlinear optical applications and spontaneous emission control^2^,^3^,^4^,^5^. These metamaterials can be described within an effective medium description by a diagonal permittivity tensor with components ε_xx_ = ε_yy_ and ε_zz_. This description is accurate if the size and distance between the nanostructures forming the metamaterial is much smaller than the wavelength and if the values of the effective permittivity components are not vanishing, where nonlocal effects occur^6^. Such metamaterials support two types of propagating waves, called ordinary and extraordinary waves. Ordinary waves have an electric field normal to the z axis. Therefore, for these waves the metamaterial has an effective permittivity ε_xx_ with a typical spheroid isofrequency surfaces. Extraordinary waves have a component of the electric field along the anisotropy axis (z-axis) and, therefore, they can have either a conventional elliptic dispersion for frequencies where both ε_xx_ and ε_zz_ have the same sign or a hyperbolic dispersion in the frequency range where ε_xx_ and ε_zz_ have opposite signs^5^. Such anisotropic metamaterials can be realized as plasmonic-dielectric multilayers with hyperbolic regime occurring for ε_xx_ = ε_yy_ < 0 and ε_zz_ > 0 or plasmonic nanorod arrays in a dielectric matrix with hyperbolic dispersion condition ε_xx_ = ε_yy_ > 0 and ε_zz_ < 0.

Such anisotropic metamaterials have recently been proposed to realise deep-subwavelength planar waveguides utilizing their peculiar hyperbolic dispersion^7^,^8^,^9^, providing a unique opportunity to guide bulk plasmon polaritons in the metamaterial slab^7^. These metamaterial-based waveguides exhibit unusual properties particularly in the case of hyperbolic dispersion where the propagating modes can be left-handed (backward waves) and highly confined^8^,^9^,^10^,^11^. While planar hyperbolic metamaterial waveguides (MWs) preserve the nature of ordinary and extraordinary modes of the infinite anisotropic metamaterial, in three-dimensional waveguides, mode confinement in 2 dimensions (2D) significantly modifies the behavior of waveguided modes of different polarizations.

In this paper, we analyze the properties of waveguided modes supported by anisotropic metamaterial waveguides with 2D confinement in both elliptic and hyperbolic regimes. It will be shown that although in some particular cases (e.g., planar waveguides) modes can be divided into extraordinary and ordinary modes^5^,^7^, in general, for arbitrary boundary conditions, eigenmodes have a mixed nature leading to hybrid modes (HMs) having properties of both extraordinary and ordinary modes. Due to the different nature of the interacting modes (conventional ordinary and backward extraordinary), hybrid modes exhibit unusual optical properties, such as anomalous dispersion leading to superluminal and stopped light, which can be used in the development of new integrated photonic devices and active metamaterial functionalities.

We consider a waveguide made of an anisotropic hyperbolic metamaterial with confinement in the direction of the extraordinary z-axis and one of the ordinary x-axis, so that modes are guided along the ordinary y-axis. In the complementary case, the confinement may be imposed in the ordinary x- and y- directions with guiding occurring along the extraordinary z-axis. The effective permittivity tensor of the metamaterial is considered the same as in the case of an infinite metamaterial: ε_xx_ = ε_yy_ > 0 and ε_zz_ < 0, corresponding to the metamaterial realization as an array of nanorods aligned along z-direction (Fig. 1(a)).

Before presenting numerical simulations of the waveguide properties, we first consider an intuitive model of an anisotropic waveguide. The coupled wave equations governing the behavior of propagating modes **E**(x, y, z) = **E**(x, z)exp(iβy) within the waveguide can be written as^12^


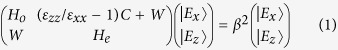


where β is the propagation constant of a given mode, k_0_ = 2π/λ_0_ and λ_0_ is the wavelength of light in vacuum, with


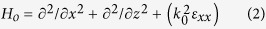



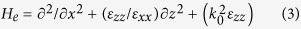



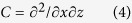


where Eqs (2) and (3) correspond to the ordinary and extraordinary waves, respectively, and W describes a coupling between modes, which takes into account boundary effects and shape and dimensions of the waveguide.

In both the planar waveguides and rectangular waveguides with perfect electric conductor (PEC) boundary conditions^13^,^14^ or with planar metamaterial waveguides^7^,^8^,^9^, W = 0. Consequently, modes with polarization along ordinary and extraordinary directions are decoupled (Fig. 1(b), first two mode profiles) and the values for the propagation constant for ordinary (β_o_) and extraordinary (β_e_) modes in Eq. (1) can be determined from


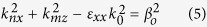



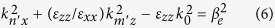


where k_nx_ = nπ/a and k_mz_ = mπ/b with (n, m) and (n’, m’) are the integers corresponding to the mode order of the ordinary and extraordinary modes, respectively, and a and b are the height and width of the rectangular metamaterial waveguide, respectively (Fig. 1(b)). Thus, when admitting the PEC boundary conditions, ordinary waveguided modes β_o_ can be decomposed choosing a basis of ordinary plane-waves, while extraordinary modes β_e_ choosing an extraordinary plane-wave basis.

In order to analyze the mode structure in different dispersion regimes, we considered the anisotropic permittivity tensor components ε_xx_ = 9 and ε_zz_ = 20 for the elliptic dispersion and ε_zz_ = −20 for the hyperbolic dispersion. These values are typical for hyperbolic metamaterials based on plasmonic nanorod arrays^15^. Figure 1(c) shows the cross-section at a given k_x_ of the isofrequency surfaces mapped by Eqs (5,6) in k-space (k_x_, β, k_z_) for both ordinary and extraordinary directions. For extraordinary modes in the elliptic regime (ε_zz_ > 0 and ε_xx_ > 0), this surface is an ellipsoid, while in the hyperbolic regime (ε_zz_ < 0 and ε_xx_ > 0), it represents a hyperboloid. Figure 1(d) shows the dispersion of ordinary and extraordinary waveguided modes in both elliptical and hyperbolic cases. While a similar behavior is observed for the ordinary (blue curve) and extraordinary elliptic (green) mode, the extraordinary hyperbolic (red) mode has a completely different behavior. In the hyperbolic case, the mode dispersion has a negative slope and, thus its group velocity is negative. Therefore, extraordinary hyperbolic modes are backward modes^5^,^16^. Ordinary and hyperbolic modes can, thus, be degenerated at frequency ω_d_ at which the propagation constants of both extraordinary and ordinary modes (in this case, the (1, 1) ordinary mode with the (1, 4) hyperbolic mode) are equal. As one can see from Eqs (5,6), the value of ω_d_ can be designed at will by changing waveguide dimensions (a, b) and metamaterial properties (ε_xx_, ε_zz_). For example, to achieve the degenerated modes at the telecom wavelength of λ_d_ = 1.55 μm, the dimensions of the waveguide should be b = 80 nm and a = 300 nm for the metamaterial parameters discussed here.

In a realistic waveguide geometry (no PEC boundary conditions), the coupling W is generally different from zero. Defining the eigenvalue equations 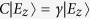, 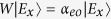, and 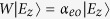, where γ and α_eo_ are the anisotropic coupling constant and the coupling constant between extraordinary and ordinary modes, respectively. The latter coupling arises from the geometrical confinement of fields in the waveguide and have magnitude governed by modal overlap between ordinary and extraordinary modes resulting from scattering at the edges of the waveguide. In this framework, the mode propagation constants in Eq. (1) take the form


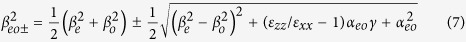


Thus, since both coupling constants are typically small, when β_e_ is significantly different than β_o_, ordinary and extraordinary waveguided modes are relatively independent with β_eo+_ ≈ β_o_ and β_eo−_ ≈ β_e_. However, when extraordinary and ordinary modes are degenerate (β_e_ ≈ β_o_), the effect of mode coupling is important, and the resulting hybrid modes β_eo_ have properties from both ordinary and extraordinary modes (e.g., in the HM mode profile in Fig. 1(b)), a situation similar to that of modes composed of skew rays propagating in circular optical fibres^13^.

It should be noted that, as can be observed from Fig. 1(c), for waveguided mode propagating along the extraordinary z-axis, it is not possible to achieve ordinary and extraordinary modes (defined by the blue and red dispersions, respectively) with the same value of β in the case of hyperbolic dispersion. Therefore, in this case there is no degeneracy between ordinary and extraordinary modes and the mode hybridisation does not occur.

The coupling between ordinary and extraordinary modes in the waveguide was analyzed through the numerical simulations of the behavior of the propagation constant in a MW surrounded by air (ε = 1). The behavior of uncoupled β_e_ and β_o_ follows approximately that of the MW analyzed above using Eqs (5,6) for PEC boundary conditions (cf. Figs 1(d) and 2). In the case of elliptical dispersion (Fig. 2(a)), the degeneracy of the (1, 2) extraordinary mode and the (1, 1) ordinary mode leads to strong coupling resulting in the modification of the mode dispersions in the shape of avoided crossing. The mode profiles simulated for different frequencies show that waveguides with heights for which there is no mode degeneracy, support well-defined extraordinary and ordinary modes, while in the vicinity of the degeneracy point, HM profile is a linear combination of the two (Fig. 2(a)).

A similar analysis in the case of hyperbolic dispersion reveals very different behavior (Fig. 2(b)). For example, strong coupling between (1, 4) extraordinary mode and (1, 1) ordinary mode does not exhibit avoided crossing of the degenerate modes, leading nevertheless to strong interaction between them. The resulting shape of the dispersion curve obtained numerically can also be obtained for the coupling between the ordinary and hyperbolic extraordinary modes using equations Eqs (5, 6, 7) with the coupling constants α_eo_ = γ = 0.01. This peculiar shape is due to the interplay between positive and negative direction of the energy flow in the interacting modes. In this case, the propagation constant of the hybrid modes may have a non-zero imaginary part even in the case of lossless material constants.

This “effective” attenuation, determined by the value of ε_zz_/ε_xx_ is a consequence of the competition between the forward waves (ordinary wave components) and the backward waves (extraordinary wave components) in the hybrid waveguided mode. Thus, the mode is allowed to take a backward path through its extraordinary component, leading to a limit in its total propagation length and to its effective attenuation even if, for the sake of the argument, lossless materials are considered.

In a multimode waveguide, multiple degeneracies of ordinary and extraordinary modes exist at different wavelengths, depending on the waveguide dimensions. The coupling strength between the modes depends on the overlap of their fields and is different for different modes. This can be observed in the variations of the effective refractive index n_eff_ = β/k_0_ of the hybrid modes with the height of the waveguide in both the elliptic and hyperbolic case (Fig. 3). For example, in Fig. 3(b), the coupling of 4 hyperbolic extraordinary modes and the ordinary mode is seen with different strength. In turn, a variation of the MW’s width will only lead to a shift of the degeneracy frequency (Eqs (5, 6, 7)). Furthermore, increasing material losses result in the decrease of the coupling strength between extraordinary and ordinary modes (Figs 3(a,b) and 4(a)). As losses increase, β_eo+_ tends to β_o_ and β_eo−_ to β_e_.

Since the hybrid modes obtained through the coupling between the extraordinary hyperbolic mode and ordinary elliptic mode exhibit a complex dispersion (Fig. 4(a)), their group velocity and group velocity dispersion have interesting properties. First, in the low loss case, we observe that the hybrid mode β_eo+_, corresponding to the forward hybrid mode, has two points of low group velocity (Fig. 4(b)). In the lossless case, the amplitudes of the peaks become infinite and the group velocity is zero, corresponding to the so-called stopped-light regime^17^,^18^. The reduction of the group velocity is strongly dependent on the losses in the hyperbolic media (red and blue curves in Fig. 4(b) but is present even for unrealistically increased losses. For the artificial hyperbolic metamaterials based on Au nanorod arrays, relevant for the consideration above, the losses can be controlled through the annealing of the electrodeposited gold^19^ and Im(ε_zz_) is of the order of 0.1 or lower. Smaller values are possible with silver nanorod arrays^20^. Furthermore, several natural hyperbolic materials, such as hexagonal boron nitride, can provide low-loss hyperbolic behavior^21^,^22^, suitable for the observation slow waveguided modes described above.

The stopped-light behavior can be intuitively understood from the competition between ordinary and extraordinary modes with opposite group velocities. Therefore, these metamaterial waveguides can be used for the design of stopped-light lasers^23^, enhancement of nonlinear effects, optical switching, quantum optics and optical data storage^18^,^24^. In particular, in the case of stopped-light lasers, a MW can be designed to achieve stopped light (or slow light considering losses) with large tuneability since the properties of the metamaterial can be designed from its geometry and composing materials, thus allowing the lasing enhancement at any chosen wavelength. Interestingly, low group velocity can also be achieved in planar hyperbolic metamaterial waveguides for pure TM modes^7^,^8^,^9^,^10^, where the phenomenon has a different nature and related purely to metamaterial dispersion. Similarly, group velocity can be engineered and controlled with loss using interplay between material and waveguide-geometry dependent dispersion in the confined waveguides with the PEC cladding, where the mode coupling is also absent^25^.

Furthermore, the spectral range of a very high group velocity is also present in the HM dispersion, corresponding to the creation of “superluminal waves”^26^. These hybrid modes have regions of very strong negative and positive group velocity dispersion (GVD) as is seen in Fig. 4(c). The largest GVD is obtained around the stopped light points, since in this regime, one of the spectral components is stopped while the others are still propagating (i.e., the spectral components of a light pulse). Such strong GVD is suitable for implementing microscale metamaterial inserts in optical fibres to achieve dispersion control for pulse reshaping in optical communications^27^.

In conclusion, the strong coupling between extraordinary and ordinary modes induced by geometrical confinement in the waveguides made of an anisotropic metamaterial has been studied analytically using first-order perturbation theory and numerical simulations. Such coupling is absent in planar hyperbolic waveguides based on layered or nanorod composites, where TE and TM modes are independent^7^,^8^,^9^. The results show a typical strong coupling behavior in the case of the metamaterial with a conventional elliptic dispersion, whereas in the case of the metamaterial with a hyperbolic dispersion, the strong coupling results in the hybrid modes with anomalous dispersion. This anomalous dispersion exhibits features such as stopped and superluminal regimes, both depending on modal losses. The related GVD shows both positive and negative regimes with large GVD value, several orders of magnitude larger than typical GVD used in, e.g., pulse compression applications. It was also found that strength of the modal coupling can be controlled through the material losses thus, opening perspectives for all-optical control of the coupling, mode dispersion, group velocity and GVD of these hybrid modes via the nonlinear response of metamaterial’s metal component^3^,^28^,^29^. Such metamaterial waveguides can be incorporated in silicon-based photonic circuitry or in optical fibres for all-optical modulation, stopped-light lasing or dispersion compensation applications for on-chip ultrashort pulse generation.

## Additional Information

**How to cite this article**: Neira, A. D. *et al.* Superluminal and stopped light due to mode coupling in confined hyperbolic metamaterial waveguides. *Sci. Rep.* **5**, 17678; doi: 10.1038/srep17678 (2015).

## Figures and Tables

**Figure 1:**
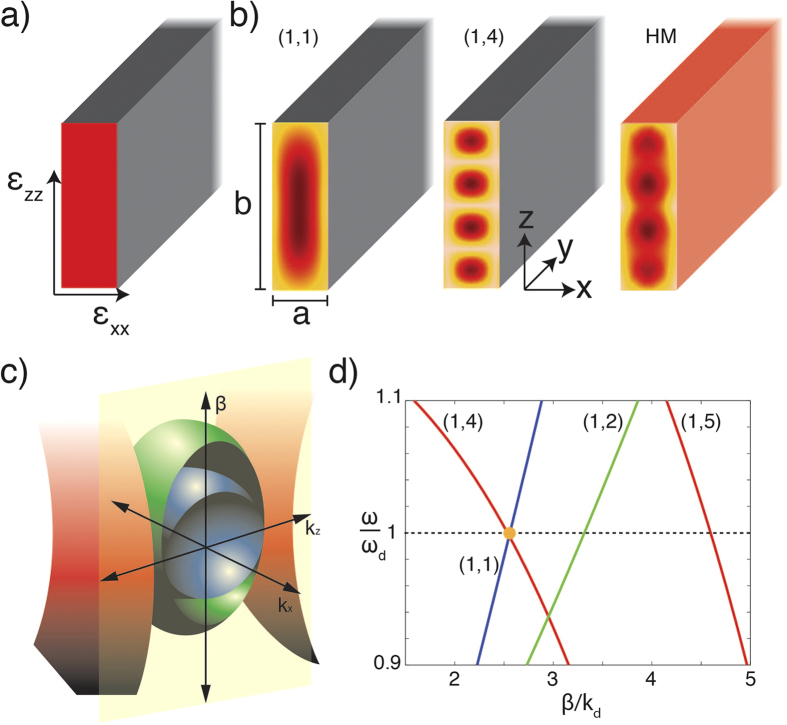
(**a**) Schematic of an anisotropic metamaterial waveguide. (**b**) (from left to right) The simulated intensity distributions for (1, 1) ordinary, (1, 4) extraordinary and hybrid modes. (1, 1) and (1, 4) modes are calculated using PEC boundary conditions, while the HM mode is calculated for a realistic MW in air. (**c**) Isofrequency surfaces for the elliptic regime (ε_xx_ = 9 and ε_zz_ = 20): (blue) ordinary and (green) extraordinary modes, and (red) extraordinary hyperbolic modes (ε_xx_ = 9 and ε_zz_ = −20). (**d**) Mode dispersions from (**c**). The colors of the lines in (**d**) corresponds to the isofrequency surfaces in (**c**) for each case.

**Figure 2:**
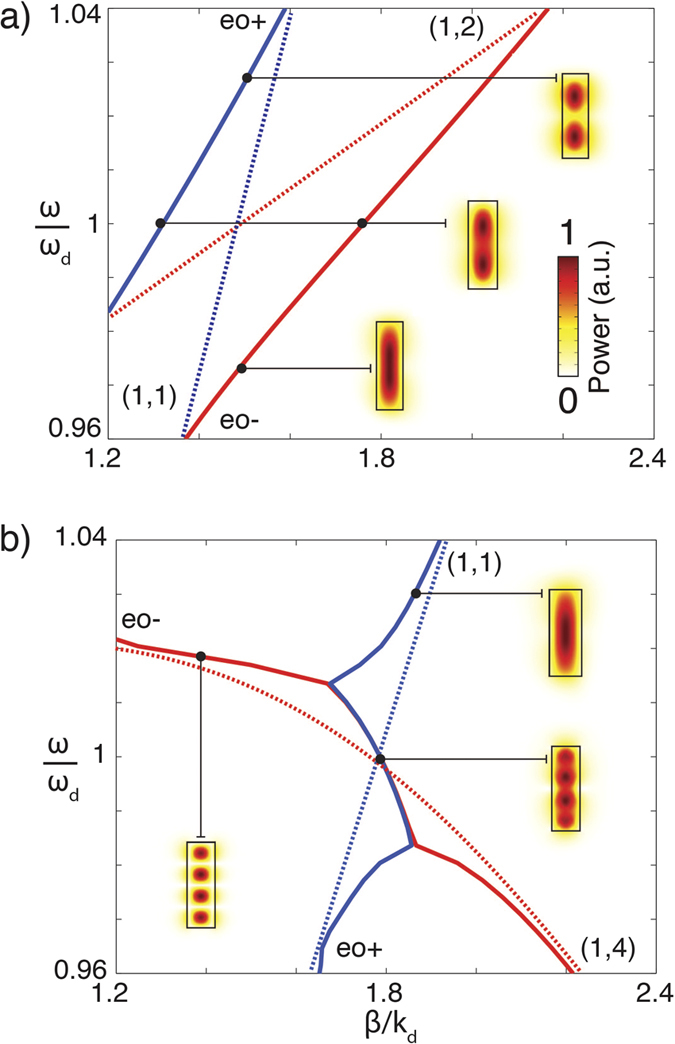
Dispersion of the waveguided modes in MW near the degeneracy points in the case of (**a**) elliptic and (**b**) hyperbolic dispersions. Solid lines are the full-vectorial numerical simulations: (red) β_eo+_ and (blue) β_eo–_. Dashed lines are the model with PEC boundary conditions where coupling is absent: (red) extraordinary and (blue) ordinary modes. The mode profiles are also shown. The metamaterial parameters are as in Fig 1.

**Figure 3:**
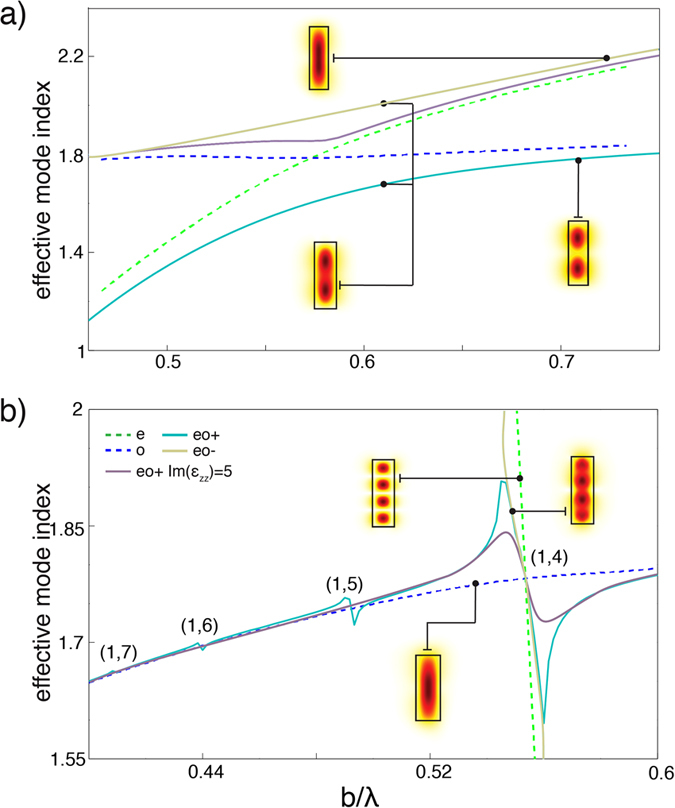
The dependence of the effective mode index on the height of the waveguide for ordinary, extraordinary and hybrid modes in the case of (**a**) elliptic and (**b**) hyperbolic dispersion regime. The modes profiles are also shown. The mode identification is shown in (**b**). The modes for the metamaterial are simulated with Im(ε_zz_) = 0.1 typical for Au-based nanorod metamaterials and with the loss increased 50 times (Im(ε_zz_) = 5). The waveguide width is fixed at a/λ = 0.3.

**Figure 4:**
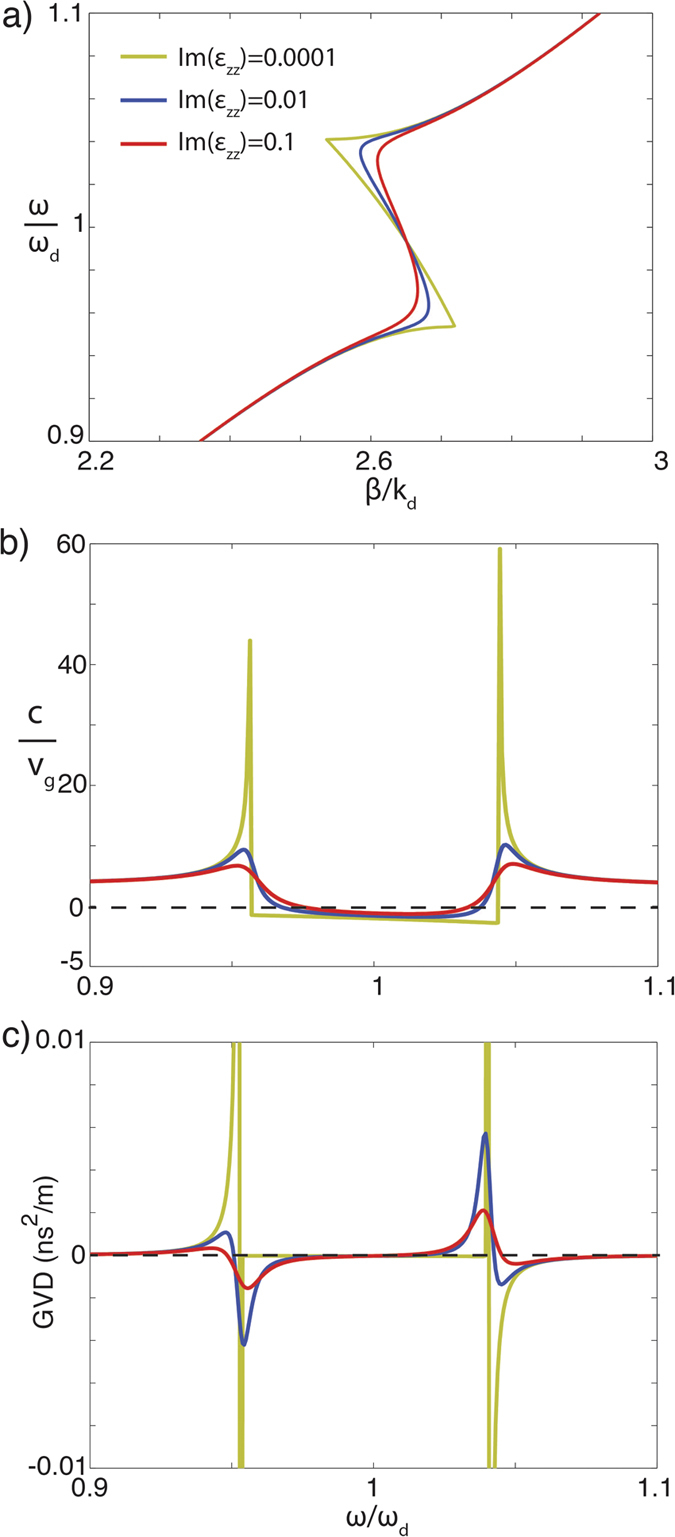
(**a**) Dispersion, (**b**) group velocity and (**c**) group velocity dispersion of the hybrid hyperbolic modes (β_eo+_) for different material losses. The same dependences can be obtained by varying the imaginary part of only ε_xx_ or ε_zz_ or of both ε_xx_ and ε_zz_ at the same time. The other waveguide parameters are the same as in Fig. 1.
